# Point-of-care viscoelastic coagulation assessment in healthy dogs during the perianesthetic period

**DOI:** 10.1186/s12917-022-03442-x

**Published:** 2022-09-14

**Authors:** Wen H. Wang, Alex M. Lynch, Julie A. Balko, Daniel J. Duffy, James B. Robertson, Lysa P. Posner

**Affiliations:** 1 Department of Molecular Biomedical Sciences, College of Veterinary Medicine, North Carolina State University, 1060 William Moore Dr, Raleigh, NC 27607 USA; 2 Department of Clinical Sciences, College of Veterinary Medicine, North Carolina State University, 1060 William Moore Dr, Raleigh, NC 27607 USA; 3 Office of Research, College of Veterinary Medicine, North Carolina State University, 1060 William Moore Dr, Raleigh, NC 27607 USA

**Keywords:** Coagulation, Viscoelastic testing, VCM Vet, Perianesthetic period

## Abstract

**Background:**

The viscoelastic coagulation monitor (VCM Vet) is a novel, portable device that provides a global assessment of hemostasis. The study aims were to evaluate serial viscoelastic analysis during the perianesthetic period in healthy dogs and to compare the agreement between two VCM Vet devices. Twenty healthy dogs undergoing orthopedic surgery were enrolled. Whole blood samples were collected from an intravenous catheter at four time points: baseline, 15 min after premedication, 60 min after inhalant initiation, and 60 min after inhalant termination. Viscoelastic tests were performed in duplicate on different devices, providing: clot time (CT; seconds), clot formation time (CFT; seconds), alpha angle (α; degrees), amplitude (units) at 10 (A10) and 20 (A20) minutes post clot time, maximum clot firmness (MCF; units), and lysis index (%) at 30 (Li30) and 45 (Li45) minutes post maximum clot formation.

**Results:**

One hundred sixty samples were analyzed. The speed of CT and CFT significantly decreased an average of 25.5 s (95% confidence interval [CI]15.9–35.0) and 6.9 s (95% CI 3.1–10.7) per time point, respectively. There were no significant changes in clot strength or lysis variables. The Bland–Altman style plot shows an acceptable rate of agreement for all variables with intra-class correlation ranging from 0.64–0.94.

**Conclusion:**

The rate of clot formation (CT and CFT) decreased over the perianesthetic period in healthy dogs undergoing surgery. These changes were small and occurred without changes in clot strength or fibrinolysis rate, thus were not clinically relevant. There was clinically acceptable consistency between devices.

**Supplementary Information:**

The online version contains supplementary material available at 10.1186/s12917-022-03442-x.

## Introduction

Monitoring the coagulation status of small animals has traditionally involved plasma-based clotting assays (prothrombin time [PT] and activated partial thromboplastin time [aPTT]), which gauge the time required for fibrin to form. These tests are best suited for detecting hypocoagulability yet are insensitive predictors of bleeding risk [1]. A more global assessment of hemostasis is possible with viscoelastic monitoring such as thromboelastography [TEG] and rotational thromboelastometry [ROTEM]). Viscoelastic testing provides data on the rate of clot formation, final clot strength, and rate of clot breakdown. Viscoelastic indicators of clot strength might predict *in-vivo* hemostatic competence more accurately in humans [2] and dogs [3]. Viscoelastic tests are commonly used to guide intraoperative transfusion administration in humans undergoing major surgeries [4, 5]. Their use in dogs during the perianesthetic period for coagulation monitoring or transfusion decisions has not yet been described.

Viscoelastic coagulation monitoring is well described in veterinary medicine [6]. However, its use has been mostly restricted to specialist centers, due to its expense and the complexity of testing [7]. A novel portable handheld viscoelastic coagulation monitor (VCM Vet) has become commercially available. In contrast to traditional viscoelastic systems, the VCM Vet is a reagent-free, cartridge-based system that uses < 300 µL of fresh whole blood and provides results in a maximum of 60 min. Normal reference ranges have been established for un-medicated healthy dogs [8]. Coagulation monitoring during the perianesthetic period is often needed due to underlying patient disease, surgical complications, intravenous (IV) fluid therapy, and/or the administration of anesthetic drugs. Although anesthetic drugs have been shown to impact changes in platelet and clotting factor function [9, 10], there is little evidence of increased bleeding events. Additionally, the effects of anesthetic drugs on coagulation are not well documented by viscoelastic tests. There is insufficient data on the effects of anesthetic drugs, surgery, and fluid therapy on coagulation in healthy dogs during the perianesthetic period. Viscoelastic testing with this point-of-care device is a viable tool to fill that knowledge gap.

The primary objective of this pilot study was to evaluate serial viscoelastic parameters of native whole blood in healthy dogs during the perianesthetic period with the VCM Vet point-of-care viscoelastic device. A secondary objective was to evaluate the agreement (reliability of results) between two VCM-Vet devices. We hypothesized that data from viscoelastic measurements obtained on the VCM Vet device would not change during the perianesthetic period in healthy dogs undergoing elective orthopedic surgery. A secondary hypothesis was that there would be clinically acceptable consistency between variables measured on different VCM Vet devices.

## Material and methods

### Study design

A prospective observational pilot study involving client-owned dogs presented to a university veterinary teaching hospital (North Carolina State University) was performed and was approved by a Teaching Hospital Clinical Protocol and Institutional Animal Care and Use Committee (protocol 19–783).

### Animals

Twenty dogs presented for elective orthopedic procedures between February and October 2020 were prospectively enrolled. Dogs were deemed to be healthy based on history, physical examination, baseline laboratory testing (packed cell volume [PCV], total protein [TP], and platelet count), and review of their drug history. Exclusion criteria included American Society of Anesthesiologists (ASA) physical status III or greater, PCV < 0.39 L/L (< 39%), age < 6 months or > 10 years, bodyweight < 15 kg, or aggressive or excitable behavior. Dogs that were administered drugs that could alter hemostasis (such as corticosteroids or antithrombotic agents) within 7 days prior to surgery were excluded, with the exception of non-steroidal anti-inflammatory drugs (NSAIDs). Dog breeds associated with hemostatic abnormalities were excluded, including Greyhounds, Cavalier King Charles spaniels, Akitas, and English bulldogs [11–15].

### Anesthesia

Anesthetic protocols, fluid administration, and loco-regional anesthesia were not standardized and were performed at the anesthetist’s discretion. Prior to pre-medication, each dog had an 18-Ga, 48 mm polyurethane intravenous catheter (BD Insyte; Covetrus) aseptically placed in a cephalic or saphenous vein by the same investigator (WHW). Each catheter was then flushed with 0.9% saline solution (Monoject Prefill IV Flush; Covidien) every 6 h prior to premedication through an extension port (Lifeshield Microbore Extension Set; Hospira). Following induction of general anesthesia, dogs were orotracheally intubated, and general anesthesia was maintained with isoflurane (Isoflurane; Piramal Critical Care) in > 95% oxygen. Capnography, 3-lead electrocardiography, pulse oximetry, non-invasive oscillometric blood pressure, and body temperature (BT ºC [Celsius]) were monitored with a multiparameter monitor (Mindray Passport 12; Mindray) and were recorded every 5 min following induction of anesthesia until extubation. Total administered fluid volume (mL) as well as any perianesthetic complications and their treatment(s) were recorded.

### Data collection

Blood was collected from the pre-placed intravenous catheter at 4 time points: after catheter placement but before administration of any anesthetic drugs (< 16 h from placement) (baseline[BL]), 15 min following premedication (PM), 60 min after inhalant initiation (A), and 60 min after inhalant termination (R). The dead space of the IV catheter and extension set was measured with an ex vivo saline displacement technique. All blood samples were collected by the same investigator (WHW) using a 3-syringe technique [16]. Briefly, an empty 3-mL plastic syringe (Monoject Luer Lock Syringes; Covidien) was attached to the extension set, and 1.2 mL of blood (300% of the calculated catheter and extension set dead space) was aspirated and discarded [17]. A new 3-mL syringe was then used to collect 1 mL of blood before flushing the catheter with 2 mL of 0.9% saline solution through the extension set.

At every time point, sampled blood was used for viscoelastic testing, determination of PCV and TP, and blood smear preparation. At baseline and 60 min after inhalant initiation, venous blood gas (VBG) analysis (VetScan iStat cG8 + Cartridge; Abaxis, Inc.), glucose (glucometer; Alpha TRAK2; Zoetis) and lactate (lactate meter; Lactate Plus; Nova Biomedical) measurements were also performed.

### Viscoelastic coagulation testing

Before performing analyses, system checks were performed per the manufacturer’s direction on each device (VCM Vet; Entegrion). Immediately after blood collection, whole blood was dispensed directly into two cartridges (VCM Vet cartridges; Entegrion) that were pre-warmed to 37 °C using the manufacturer’s heat plate. Once the cartridge reservoir was full, they were placed into one of two devices, and testing was then initiated and run for the total duration of the test (60 min). Samples were run in duplicate at each time point, with the order of machines alternated for successive time points.

The following variables were determined: clot time (CT), clot formation time (CFT), alpha angle (α), amplitude at 10 and 20 min post clot time (A10 and A20, respectively), maximum clot firmness (MCF), and lysis index at 30 (Li30) and 45 (Li45) minutes indicating the amplitude of the clot 30 and 45 min after clot time as a percentage of MCF respectively. After all data were collected (Additional file 1: Appendix B), the viscoelastic tracings were anonymized and then individually reviewed by one author (AML). Tracings were evaluated following a rubric (Additional file 1: Appendix A) with the rate of clot formation, clot strength, and rate of clot lysis stratified as being increased, normal, or decreased from interpretation of the numeric value for each device variable. Secondly, an overall interpretation (hypercoagulable, normocoagulable, hypocoagulable, or hyperfibrinolytic) was then assigned based on the results of these three aforementioned components.

Surplus blood collected at each time point was placed into a plastic tube containing potassium EDTA (Monovette 1.2 mL; Sarstedt Inc.) for subsequent determination of PCV, TP and platelet count (PLT). PCV was measured via centrifuged, non-heparinized micro-hematocrit tubes (Fisher Scientific; Pittsburgh), TP was determined using a refractometer, and platelet count was estimated from blood smear evaluation. Blood smears from each time point were made within 30 min of blood collection. Once dried, blood smear slides were stained with a rapid stain (Diff-Quick; JorVet) and examined by the same investigator (WHW) under the direct supervision of a board-certified criticalist (AML). The platelet count (/µL) was estimated using a previously validated technique by multiplying the average number of platelets seen over 10 high power fields (100x) by 20,000 [18].

### Statistical analysis

Continuous variables were assessed for normality using the Shapiro–Wilk test alongside graphical summaries. Where appropriate, data were reported as the median and interquartile range (IQR) or mean and standard deviation (SD). Viscoelastic variables from each device at each time point were compared and analyzed using linear mixed models with random intercepts for individual patients. Analyzing the viscoelastic variables through time, each model consisted of one variable as the response and time point and device as predictors. For the variables MCF and α angle, a simple paired *t* test was powered to detect a mean difference of at least 5 units (85.7%) and at least 5 degrees (80.5%), respectively. These calculations were based on observed standard deviations, using the pair of smallest standard deviations to determine the subtlest detectable difference. The relationship between anesthesia duration and changes in CT and CFT was analyzed using multiple linear mixed models with a random intercept for patient. Differences in PCV, TP, and BT between time points were analyzed with paired *t*-tests and a Wilcoxon signed-rank test was used for VBG variables and PLT which were not normally distributed. Significance was set at a *P*-value of < 0.05, although Bonferroni corrections were used for variables that had multiple comparisons. Specifically, *P* < 0.00625 when comparing viscoelastic variables, and values of *P* < 0.00357 for comparing VBG variables at baseline and 60 min after inhalant initiation. A value of *P* < 0.0167 was considered significant when comparing if PCV, TP, BT, and PLT changed over time.

Agreement between the point-of-care devices was assessed by using Bland–Altman (BA) style analyses for all time points. A visual display of agreement between paired samples was used for BA style analyses, the outliers were identified for removal based on visual inspection of residuals (whose mean values were visually separated from the bulk of the data) and evaluations of Cook's distances (CFT > 350 s, Li30 < 95%, Li45 < 90%). The intra-class correlation coefficient (ICC) for agreement under a two-way model was calculated as well for each variable. Results of the BA analyses and ICC were then reviewed for each pair of variables to determine if there was clinically acceptable consistency between devices (i.e. the authors evaluated whether the same variable derived from the two devices were concordant with each other). Statistical analyses were performed using R Version 4.0.3 (The R Foundation, https://www.r-project.org) with packages *lmer, lmerTest*, and *irr*.

## Results

### Study population

Twenty dogs (11 castrated males and 9 spayed females) were enrolled. The median (IQR) age and weight were 2.4 (0.8—8.2) years and 36.8 (26.9—44.2) kg, respectively. Eleven breeds were represented, consisting of Labrador Retriever (*n* = 6), Beagle (*n* = 2), Great Dane (*n* = 2), Labradoodle (*n* = 2), and Mastiff (*n* = 2). One Cane Corso, Doberman pinscher, German Shepherd, Great Pyrenees, mixed-breed dog, and Rhodesian ridgeback. Surgical procedures included: tibial plateau leveling osteotomy (*n* = 8), bilateral elbow arthroscopy (*n* = 3), and one each of unilateral tarsal arthroscopy, pantarsal arthrodesis, triple tibial osteotomy, femoral head, and neck osteotomy, common calcaneal primary tendon repair, cranial closing wedge osteotomy, correction of medial patella luxation, tibial deformity correction, and partial medial meniscectomy.

### Anesthesia management

General anesthesia was induced with ketamine (range 1–2 mg/kg, Ketaset 100 mg/mL; Zoetis Inc.) and propofol (range 1–3 mg/kg, Propofol, 10 mg/mL; Sargent Pharmaceuticals) in all dogs. Intravenous crystalloid (Lactated Ringer’s solution; B. Braun Med.) was administered at a rate of 3–5 mL/kg/hr. Premedication, intra-operative treatments, and adjunct analgesia are listed in Table 1. The duration of anesthesia, surgery and time from baseline to 15 min following premedication are listed in Table 1. Physiologic variables, PCV, TP, PLT, and intravenous fluid amounts at each time point are listed in Table 2. Excessive platelet clumping was noted in 8 samples (*n* = 6 dogs) that precluded obtaining accurate platelet counts (3 samples at 60 min after inhalant initiation, and 5 samples at 60 min after inhalant termination).

**Table 1:** Summary of premedication, intraoperative treatment, adjunct analgesics, surgery and anaesthesia duration in dogs undergoing anaesthesia and elective orthopaedic surgery (*n* = 20). Data are expressed as median (interquartile range; IQR); animal number (*n)* and percentage (%)

**Locoregional techniques *n* (%)**	19 (95%)
**Premedication:**	
Maropitant (1 mg/kg) *n* (%)	6 (30%)
Maropitant (1 mg/kg) and Ondansetron (0.5 mg/kg) *n* (%)	11 (55%)
Hydromorphone (0.1 mg/kg) *n* (%)	6 (30%)
Dexmedetomidine (1 mcg/kg) and Hydromorphone (0.1 mg/kg) *n* (%)	12 (60%)
Dexmedetomidine (1 mcg/kg) and Fentanyl (3 mcg/kg) *n* (%)	1 (5%)
Dexmedetomidine (1 mcg/kg) and Methadone (0.3 mg/kg) and Ketamine (1 mg/kg) *n* (%)	1 (5%)
**Intraoperative anaesthetic management:**	
Fluid bolus *n* (%) (> 5 mL/kg / < 5 mL/kg)	3 (15%) / 4 (20%)
Glycopyrrolate *n* (%)	9 (45%)
Norepinephrine *n* (%)	2 (10%)
**Intraoperative adjuvants:**	
Ketamine CRI *n* (%)	13 (65%)
Dexmedetomidine CRI *n* (%)	3 (15%)
Fentanyl CRI *n* (%)	1 (5%)
Lidocaine CRI *n* (%)	1 (5%)
**Postoperative carprofen administration *n* (%)**	15 (75%)
**Use of gabapentin prior surgery *n* (%)**	4 (20%)
**Use of carprofen within 7 days prior surgery *n* (%)**	6 (30%)
**Received carprofen subcutaneously prior surgery *n* (%)**	6 (30%)
**Anaesthesia duration (minutes)**	270 (IQR:245–336 min)
**Surgery duration (minutes)**	127 (IQR:89–163 min)
**Time between BL and PM (minutes)**	810 (IQR:696–1080 min)

*CRI* Continuous rate infusion, *BL* Baseline, *PM* = 15 min following premedication

**Table 2:** Median (IQR) heart rate (HR), respiratory rate (*f*R), body temperature (BT), end-tidal partial pressure of carbon dioxide (P_E_’CO_2_), peripheral capillary oxygen haemoglobin saturation (SpO_2_), mean arterial pressure (MAP) and pack cell volume (PCV), total solids (TS), manual platelet counts (PLT) and fluid volume of 20 dogs undergoing anaesthesia and elective orthopaedic surgery. Study stages presented as BL = baseline, PM = 15 min following premedication, A = 60 min after inhalant initiation, R = 60 min after inhalant termination

	**Time points**			
**Parameter**	**BL**	**PM**	**A**	**R**
**HR (beats/minute)**^a^	92 (80–105)	67 (46–80)	63 (45–75)	80 (80–91)
**fR (breaths/minute)**	30 (20–41)	18 (12–28)	8.5 (6–10)	20 (16–39)
**BT(Cº)**^a^	37.9 (37.7–38.5)	38.0 (37.4–38.5)	36.5 (35.8–37.1)^b^	36.9 (36.4–37.3)^b^
**P**_**E**_**´CO**_**2**_** (mmHg)**	n/a	n/a	45 (38–47)	n/a
**SpO**_**2**_** (%)**	n/a	n/a	99 (98–99)	n/a
**MAP (mmHg)**^a^	94 (84–117)	90 (82–99)	69 (61–82)	96 (81–112)
**PCV (SI)**^a^	0.45 (0.42- 0.48)	0.43 (0.4–0.45)	0.38 (0.34–0.4)^b^	0.4 (0.37- 0.42)^b^
**TS (g/dL)**^a^	6.6 (6.4- 6.9)	6.6 (6.3–7.0)	6.0 (5.9–6.4)^b^	6.2 (5.9–6.5)^b^
**PLT (10**^**3**^** µL)**	196 (127–242)	155 (120- 202)	142 (128–188)	165 (150–202)
**Fluid (mL)**	0	0	108 (38–224)	556 (334–734)

*n/a* Not available

^a^ Normally distributed data

^b^ Value differs significantly (*P* < 0.001) compared with time point baseline (BL)

### Hemostatic variables

**Fig. 1:**
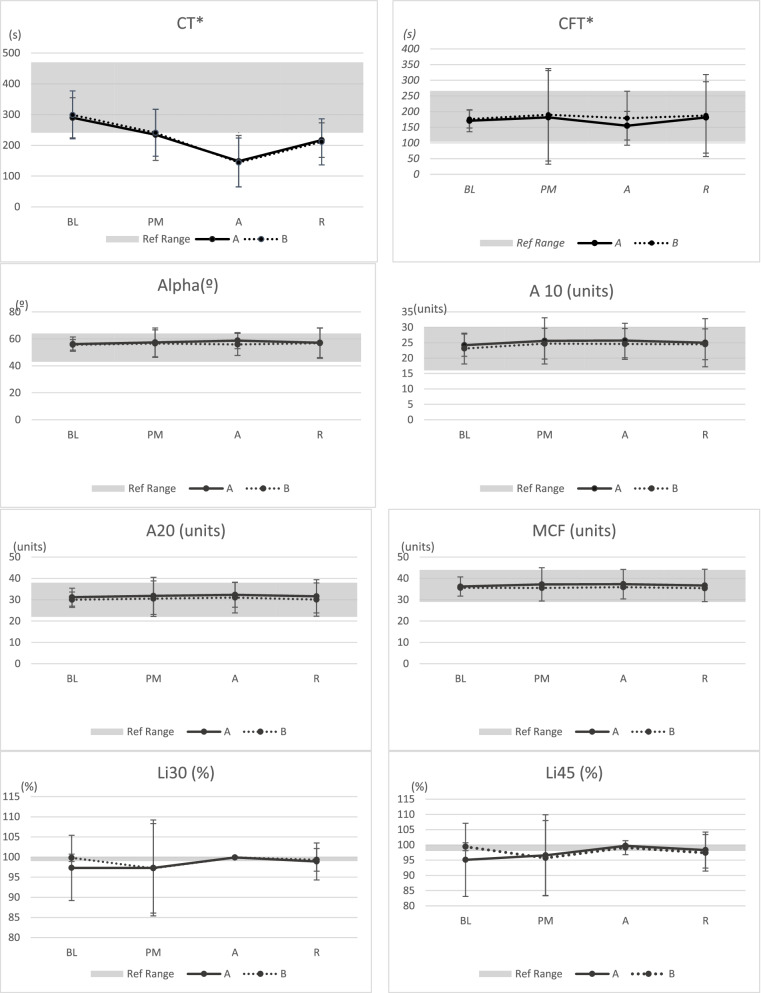
Mean ± standard deviation Viscoelastic Coagulation Monitor Vet (VCM Vet) variables from 2 devices (A and B) from 20 dogs undergoing anaesthesia and elective orthopaedic surgery. Study stages presented as BL = baseline, PM = 15 min following premedication, A = 60 min after inhalant initiation R = 60 min after inhalant termination in x-axis. Measurements units presented in y-axis. References range for each variable are illustrated as shaded area. Abbreviations: CT = clot time; CFT = clot formation time; Alpha = alpha-angle; A10 and A20 = amplitude at 10 and 20 min, respectively; MCF = maximum clot firmness; Li30 and Li45 = Lysis Index at 30 and 45 min, respectively. S = seconds, º = degrees, Units = VCM units, % = percentage. * CT decreasing an average of 25.5 s per time point and CFT decreasing an average of 6.9 s per time point (*P* < 0.001)

In total, 160 individual samples were collected from 20 dogs. No system check errors were encountered for either device. Mean ± standard deviation of viscoelastic variables are presented in Fig. 1. Visual evaluation of tracings by the blinded investigator are presented in Table 3. There was a significant decrease in CT and CFT over time, with CT decreasing an average of 25.5 s per time point [95% confidence interval (CI): 15.9–35.0 s (*P* < 0.001)] and CFT decreasing an average of 6.9 s per time point [95% CI: 3.1–10.7 s (*P* < 0.001)]. There were no significant changes in any other viscoelastic variables over time. All viscoelastic variables were within the reference interval for dogs with the exception of the mean CT and lysis index. Mean CT was outside of the reference interval at two-time points (60 min after inhalant initiation and 60 min after inhalant termination). Mean lysis index (Li30 and Li45) was also outside of the reference interval at two-time points (baseline and 15 min following premedication) (Fig. 1).

**Table 3:** Summary results of Viscoelastic Coagulation Monitor Vet (VCM Vet) tracings (*n* = 160) from 20 dogs undergoing anaesthesia and elective orthopaedic surgery over the four study stages presented as BL = baseline, PM = 15 min following premedication, A = 60 min after inhalant initiation, R = 60 min after inhalant termination, showing the number (%) of tracings with decreased, normal, or increased clot formation rate, clot strength, and clot lysis rate and overall interpretation of number (%) of tracings that were hypocoagulable, normocoaguble, hypercoagulable, or hyperfibrinolytic. All tracings evaluated following a rubric (Additional file 1: Appendix A) by single blinded investigator

**Trace interpretation**		**BL**	**PM**	**A**	**R**
**Clot formation rate *n* (%)**	Decreased	7 (17.5)	20 (50)	35 (87.5)	31 (77.5)
	Normal	33 (82.5)	20 (50)	5 (12.5)	9 (22.5)
	Increased	0 (0)	0 (0)	0 (0)	0 (0)
**Clot strength *n* (%)**	Decreased	0 (0)	4 (10)	2 (5)	4 (10)
	Normal	39 (97.5)	30 (75)	30 (75)	34 (85)
	Increased	1 (2.5)	6 (15)	8 (20)	2 (5)
**Clot lysis rate *n* (%)**	Decreased	0 (0)	0 (0)	0 (0)	0 (0)
	Normal	36 (90)	37 (92.5)	40 (100)	38 (95)
	Increased	4 (10)	3 (7.5)	0 (0)	2 (5)
**Overall** **Interpretation**	**Hypocoagulable *n* (%)**	0 (0)	2 (5)	2 (5)	4 (10)
	**Normocoagulable *n* (%)**	38 (95)	31 (78)	32 (80)	32 (80)
	**Hypercoagulable *n* (%)**	1 (2.5)	6 (15)	6 (15)	2 (5)
	**Hyperfibrinolytic *n* (%)**	1 (2.5)	1 (2.5)	0	2 (5)

### Agreement of two point-of-care devices

**Fig. 2:**
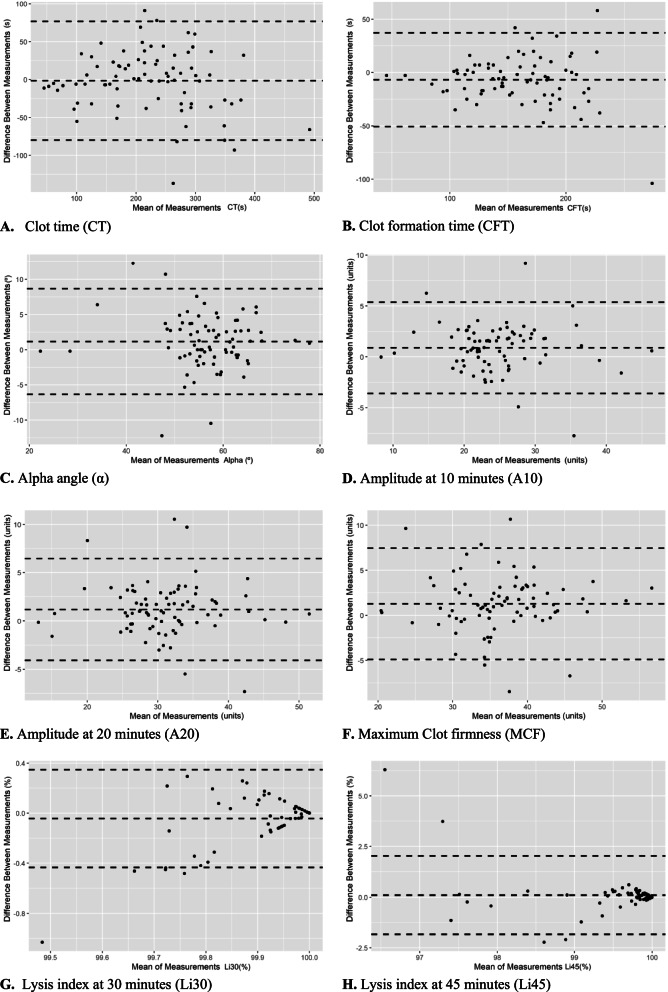
Bland–Altman plot style for comparison of paired samples from Viscoelastic Coagulation Monitor Vet (VCM Vet) variables in 20 dogs undergoing anaesthesia and elective orthopaedic surgery over four study stages (BL = baseline, PM = 15 min following premedication, A = 60 min after inhalant initiation R = 60 min after inhalant termination; different time points are not marked differently). Middle black dash line = mean difference between paired samples; upper and lower black dash line = limits of agreement. Abbreviations: s = seconds; º = degrees; Units = VCM units; % = percentage

Agreement between devices is demonstrated via Bland–Altman style plots in Fig. 2. The interval of limit of agreements for all variables were reviewed and considered clinically acceptable (i.e. data obtained from the two devices were consistent with the same clinical interpretation of being above, within, or below the reference interval for that variable). The ICC for each viscoelastic variable are reported as their ICC and 95% CI as follow: CT = 0.90 (0.85–0.94), CFT = 0.94 (0.90–0.96), α angle = 0.89 (0.83–0.93), A10 = 0.89 (0.87–0.95), A20 = 0.90 (0.83–0.95), MCF = 0.87 (0.79–0.88), Li30 = 0.83 (0.74–0.88) and Li 45 = 0.64 (0.50–0.76).

### Blood gas and hematological variables

Measured venous blood gas and hematologic variables are listed in Table 4. The variables pH, PCV, and hemoglobin were significantly lower at 60 min after inhalant initiation compared to baseline. The venous partial pressure of carbon dioxide (PvCO2) and total carbon dioxide (TCO_2_) were significantly higher at 60 min after inhalant initiation than compared to baseline. There were no significant differences in base excess (BE), potassium, ionized calcium, glucose or lactate between two-time points (Table 4). The variables outside of the reference intervals at 60 min after inhalant initiation were pH, PCV, and PvCO_2_ (Table 4). PCV, TP and BT were significantly decreased an average of 0.82 L/L (95% CI = 0.66–0.98), 0.62 g /dL (95% CI = 0.40–0.84) and 1.67 ºC (95% CI = 1.24–2.09), respectively, at 60 min after inhalant initiation and an average of 0.53 L/L (95% CI = 0.35–0.71), 0.53 g/dL (95% CI = 0.28–0.88) and 2.3 ºC (95% CI = 1.77–2.82), respectively, at 60 min after inhalant termination (Table 2) (*P* < 0.001).

**Table 4:** Mean (± standard deviation) venous blood gas results at two time points (BL = baseline; A = 60 min after inhalant initiation) of 20 dogs undergoing anaesthesia and elective orthopaedic surgery

**Variable**	**Units**	**Reference Range**	**Time points**		**Median Difference**	***P* value**
		**Venous**	**BL**	**A**	**BL-A**	
**pH**		7.31–7.48	7.35 ± 0.05	7.26 ± 0.05^a^	0.1	< 0.001
**PvCO2**	mmHg	27.8–47.2	40.0 ± 7.7	55.0 ± 8.3^a^	-16.7	< 0.001
**PvO2**	mmHg		50.5 ± 7.8	144.0 ± 101.4^a^	-56.0	< 0.001
**BE**	mmol/L	(-5)-(0)	-3.6 ± 2.9	-2.6 ± 2.7	-1	0.01
**HCO3**	mmol/L	18.3–26.4	22.1 ± 2.9	24.5 ± 2.3^a^	-2.9	< 0.001
**TCO2**	mmol/L	19–28	23.3 ± 3.1	26.2 ± 2.6^a^	-3	< 0.001
**sO2**	%		82.3 ± 7.2	95.3 ± 5.3^a^	-12	< 0.001
**Na + **	mmol/L	138–146	144.9 ± 2.6	143.7 ± 2.6	1	0.07
**K + **	mmol/L	3.4–4.7	4.0 ± 0.4	4.1 ± 0.4	0.1	0.57
**iCa**	mmol/L	1.19–1.40	1.4 ± 0.1	1.5 ± 0.1^a^	-0.1	0.07
**Glu**	mg/dL	74–123	109 ± 24	126 ± 27	-6	0.06
**PCV**	SI	0.39–0.58	44.6 ± 3.3	35.4 ± 3.9^a^	0	< 0.001
**Hb**	g/dL	12–19	14.9 ± 1.6	11.3 ± 1.2^a^	3.4	< 0.001
**Lac**	mmol/L	0.6–2.9	1.3 ± 0.5	1.1 ± 0.5	0.2	< 0.001

^a^ Value differs significantly (*P* < 0.001) compared with time point baseline (BL)

## Discussion

Compared with baseline values, healthy dogs during the perianesthetic period demonstrated a more rapid clot rate (CT and CFT), while clot strength and lysis indicators were unchanged. While the changes in clot rate time were small and unlikely to be clinically relevant, they refute the hypothesis that viscoelastic coagulation variables would be unaffected by anesthesia and surgery. This study also demonstrated clinically acceptable agreement for variables obtained on two different point-of-care devices supportive of our initial hypothesis.

Viscoelastic tests provide additional insight into hemostatic function compared with traditional plasma-based tests such as PT and aPTT. Traditional tests are most useful to assess hypocoagulability, while viscoelastic tests can identify hypocoagulability, hypercoagulability, and hyperfibrinolysis [6]. The rate of clot formation is determined by clotting factors and platelet function and is represented by the variables CT, CFT, and α angle on this device. Clot strength is determined by fibrinogen content and platelet number; moreover, it is best represented by the viscoelastic variable MCF. MCF is similar to maximum amplitude (MA) on TEG, which has been used to predict bleeding tendency in humans and dogs [2, 3, 19]. Plasmin mediated fibrinolysis is evaluated by the percentage of clot breakdown 30 and 45 min after MCF, represented by Li30 and Li45 on the device.

Although the viscoelastic testing method has been developed over decades, its application as point-of-care instrumentation in surgical and trauma settings is relatively recent. Short turnaround times and real-time results delivery provide clinicians with a relevant and rapid global assessment of hemostasis. Many studies in human medicine have reported the use of various viscoelastic methods in managing perioperative bleeding and have developed transfusion protocols for specific blood products and pharmacological interventions based on testing results [20–22]. This study set out to evaluate whether perianesthestic management practices might impact VCM Vet variables in a population of healthy dogs, not anticipated to have coagulation disturbances or significant intraoperative blood loss. This information provides a foundation for next evaluating these devices in populations of animals where coagulation disturbances or bleeding might be encountered, and where viscoelastic monitoring could be useful to guide case management.

The increased rate of clot formation present at all time points after baseline was unexpected and its clinical relevance should be interpreted with caution. A recent study shows that the coagulation assessed by ROTEM in healthy beagles was minimally affected by general anesthesia [20]. Potential reasons for accelerated clotting rate (hypercoagulability) include activation of clotting factors due to presence of an in situ IV catheter or from catecholamine-induced platelet aggregation. None of the anesthetic drugs administered in the current study have been implicated in causing hypercoagulability. Intravenous catheters were pre-placed in each dog to minimize patient discomfort and avoid negatively impacting hemostatic test results associated with traumatic venipuncture. Vessel trauma associated with direct venipuncture leads to local tissue factor release that may initiate *in vitro* coagulation and promote a prothrombotic tendency [21]. Long-term presence of an intravenous catheter might also promote hypercoagulability associated with ongoing blood vessel trauma, local clot formation, and alterations in shear stress [22, 23]. In veterinary patients, traditional hemostatic tests (PT and APTT) demonstrated minimal difference when obtained from peripheral intravenous catheters maintained for < 48 h compared with direct venipuncture [24, 25]. However, in dogs, viscoelastic testing via TEG, did demonstrate shortened clotting times from blood collected from catheters compared with direct venipuncture [26]. A 3-syringe technique was utilized for blood sampling to minimize tissue factor release and clot formation in the sampled blood. The goal was to remove any released tissue factor in the waste sample, but this may not have been sufficient to prevent procoagulation effects. Further study comparing different approaches to serial blood sampling for point-of-care viscoelastic device analysis is warranted.

There is important cross-talk between inflammation and coagulation, such that pro-inflammatory states promote thrombosis [27]. While activation of coagulation by surgery as an inflammatory stimulus likely occurred, this does not explain the changes in viscoelastic variables following premedication but before anesthesia and surgery. Similarly, prothrombotic tendencies have been described in humans recovering from anesthesia, associated with catecholamine-induced platelet aggregation [10, 28]. However, since the decrease in clotting time occurred at all time points after baseline, this is unlikely the cause.

Hypercoagulability is most commonly recognized by either increased clot strength or a combination of faster clot rate with increased clot strength [29, 30]. In our study, while clot rate (CT and CFT) were significantly decreased over the perianesthetic period, no significant changes in clot strength (MCF) were not observed. Based on the overall.

In addition, pre-analytical and analytical factors may impact viscoelastic test results. Clotting rate tests on TEG and ROTEM are prone to variability [30]; however, no evidence exists for this point-of-care device regarding specific parts of coagulation (clot formation, clot strength, clot lysis). Interestingly, the abnormal rate of clot formation was the most frequent abnormality noted throughout the four study stages, occurring in 17.5%, 50%, 87.5%, and 77.5% of tracings reviewed, respectively. The risk of variability may be higher with this device since it does not utilize hemostatic activators, which have been associated with lowering the inherent variability of TEG and ROTEM [31]. A standardized blood collection, sample handling, and test protocol were used in this study to minimize the effects of pre-analytical and analytical factors. No system check issues were encountered with the device throughout the study, and all tests were started within 4 min of sample collection.

Sedatives and anesthetics have been implicated in alterations in hemostasis with ketamine and volatile anesthetics inhibiting platelet aggregation [32, 33]. A standardized general anesthesia protocol was not mandated in the study, since we did not intend to restrict our evaluation to a specific combination of drugs. Anesthesia protocols were devised and implemented at the discretion of the attending anesthesiologist. In practice, despite this this population of dogs undergoing elective orthopedic procedures were administered similar induction agents and fluid therapy. Both ketamine and isoflurane were administered to all dogs in this study, but there was no indication that either drug produced clinically relevant changes to platelet function. While discrete platelet function testing was not performed in the current study, clinically relevant platelet dysfunction might lead to viscoelastic changes consistent with hypocoagulability that were not identified in this study.

Other factors that might impact hemostasis during the perianesthetic period include administrating pharmaceuticals (synthetic IV fluids, blood products, NSAIDs), body temperature, acid–base and electrolyte disturbances, and/or blood loss. Many dogs enrolled in this study were administered NSAIDs. Oral administration of NSAIDs has been associated with changes in *ex vivo* platelet function in dogs, which could translate into increased bleeding tendency [34]. However, the viscoelastic data collected from the present study did not support the development of hypocoagulability.

Liberal synthetic fluid administration, hypothermia, and acidemia have been associated with hypocoagulability and increased bleeding risk during anesthesia [35–37]. These risk factors rarely occurred in the study population; one dog experienced moderate hypothermia (34.1ºC), three dogs were administered a 10 mL/kg crystalloid fluid bolus to manage hypotension (MAP < 60 mmHg), and although pH significantly decreased after baseline, mean pH was still within the reference range. In the study, the temperature correction was not applied in the blood gas measurements. Overall, the literature suggests that in almost all circumstance, there is no clinical advantage to using values other than those at 37ºC [38, 39]. As with platelet function, derangement of these factors could translate into hypocoagulation which was not observed in the study dogs.

In the current study, serial PCV and TP, platelet count, and blood gas analyses were measured to evaluate any potential impact on viscoelastic testing results. There was a significant decrease in PCV and TP during the A and R stages of the study. The relative reduction in PCV and TP are likely due to IV fluid administration coupled with translocation of fluids into the intravascular system from changes in systemic vascular resistance. Low circulating red blood cell mass has been postulated to cause artefactual increases in indicators of clot strength [40],although other work has suggested that plasma protein content plays a more critical role in imparting changes on viscoelastic tracings [41, 42]. Despite changes in PCV, no apparent changes in MCF were noted in the current study. The estimated platelet count was also stable throughout anesthesia. Platelet number is associate with clot strength; therefore, preservation of a stable platelet count appears consistent with the stable MCF noted throughout the study.

This study set out to evaluate this point of care viscoelastic device in a population of dogs not anticipated to have pre-existing coagulation disturbances that were undergo surgical procedures not predicted to result in notable intraoperative hemorrhage. We intended to investigate whether the perianesthestic period impacted viscoelastic variables in these dogs. This study provides a foundation upon which further work can be done focusing on animals with hemostatic dysfunction undergoing surgical procedures where bleeding might results (e.g. liver biopsy for hepatopathy). Thus, the second goal of this study was to evaluate the consistency between two point-of-care devices. In the operating environment, the blood samples drawn rarely adhere to the ideal conditions, which might introduce variability in the measurements that were not be found when the manufacturers developed the reference ranges. In our study, the overall ICC for VCM variables from duplicated measurements ranged from 0.64 to 0.94 [39, 40]. There was clinically acceptable agreement between devices based on interpretation of the BA style analyses and ICC values. There is limited information on the reproducibility of VCM Vet devices to date. Calculating the allowable total error was not feasible in this study as we were unable to make comparisons with a gold standard analysis. Although one study reported inter-device variability in VCM Vet in awake dogs [8], the investigators noted some samples had to be rerun due to cartridge filling or sampling errors. In the current study, a single investigator performed all the sample procedures. All samples were run within 4 min of collection and no technical errors (e.g., inadequately filled cartridges). These dissimilarities potentially explain the differences between the results of the two studies.

The current study has several limitations. This includes the use of a fairly homogeneous patient population that was limited to a small number of healthy medium-large breed dogs within a narrow age range. It may not be possible to extrapolate this data to dogs outside of the specific inclusion criteria. Previous studies have also demonstrated that age and sex can both impact hemostatic variables [43, 44], which could impart variation in viscoelastic variables in an individual animal. An alternative approach would have been to obtain samples from awake dogs through an IV catheter as a control population to verify any changes noted overtime were associated with the perianesthetic period. More comprehensive hemostatic profiling was not performed in these dogs (e.g. platelet function testing, individual clotting factor analysis) that may have provided greater insight into the reasons for viscoelastic changes noted in this study.

Moreover, the combination of all clinical variables (i.e. anesthetic agents, fluid therapy, duration of anesthesia, surgical manipulation) could contribute to the viscoelastic variable data obtained in the study dogs. Unfortunately, it is not possible to determine any causal relationship from our study design. An estimated platelet count was also performed rather than using an automated hematology analyzer. There is a risk of pseudothrombocytopenia secondary to platelet aggregation when using automated machines; thus, blood smear evaluation is recommended to assess for aggregation. Unfortunately, several dogs had evidence of excessive platelet clumping meaning accurate counts could not be obtained at several time points. The reference intervals used in the current study were those provided by the manufacturers, but the development of institution specific reference ranges should be considered.

## Conclusion

Point-of-care viscoelastic variables for rate of clot formation (CT and CFT) decreased over the perianesthetic period in healthy dogs undergoing anesthesia and surgery. The changes in rate of clot formation (CT and CFT) were small and occurred without changes in clot strength or fibrinolysis rate, thus they were not considered clinically relevant. Measurement differences between the two point-of-care devices were consistently within clinically acceptable limits.

## Supplementary Information

**Additional file 1: Appendix A.** Rubric consisting of representative Viscoelastic Coagulation Monitor Vet (VCM Vet) tracings for hypercoagulability, normocoagulability, hypocoagulability, and hyperfibrinolysis in healthy 20 dogs undergoing anesthesia and elective orthopedic surgery. **Appendix B.** Flow diagram on data collection process.

## Data Availability

The datasets used and/or analyzed in the current study are available from the corresponding author on reasonable request.

## Abbreviations

A: 60 Minutes after inhalant initiation
A10/20: Amplitude at 10/20 min
α: Alpha angle
BL: Baseline
BT: Body temperature
CT: Clot time
CFT: Clot formation time
IV: Intravenous
Li 30/45: Lysis index at 30/45 min
MAP: Mean arterial pressure
MCF: Maximum clot firmness
PCV: Packed cell volume
PLT: Platelet count
PM: 15 minutes following premedication
R: 60 Minutes after inhalant termination
TP: Total Protein
VCM Vet: Viscoelastic Coagulation Monitor
VBG: Venous blood gas
