# Supplementary material for: Point-of-care viscoelastic coagulation assessment in healthy dogs during the perianesthetic period
Source: BMC Vet Res. 2022 Sep 14;18:346. doi: 10.1186/s12917-022-03442-x (PMC9472389; doi:10.1186/s12917-022-03442-x)

Hypercoagulable

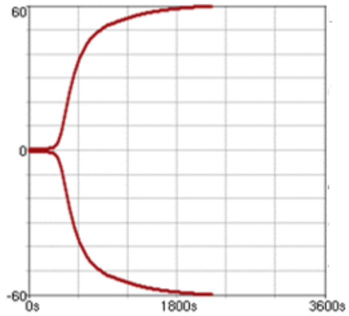

Normocoagulable

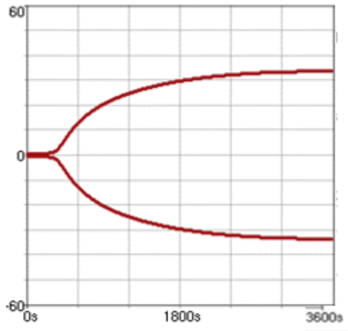

Hypocoagulable

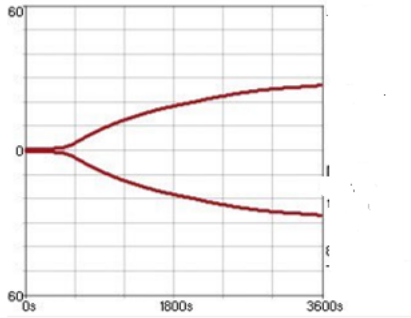

Hyperfibrinolytic

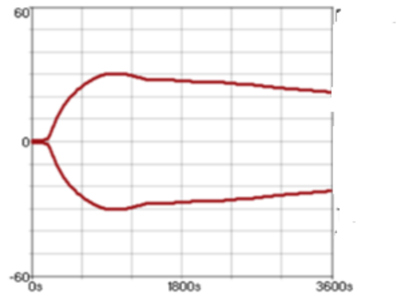

Appendix A. Rubric consisting of representative Viscoelastic Coagulation Monitor Vet (VCM Vet) tracings for hypercoagulability, normocoagulability, hypocoagulability, and hyperfibrinolysis in healthy 20 dogs undergoing anesthesia and elective orthopedic surgery.

Appendix B. Flow diagram on data collection process

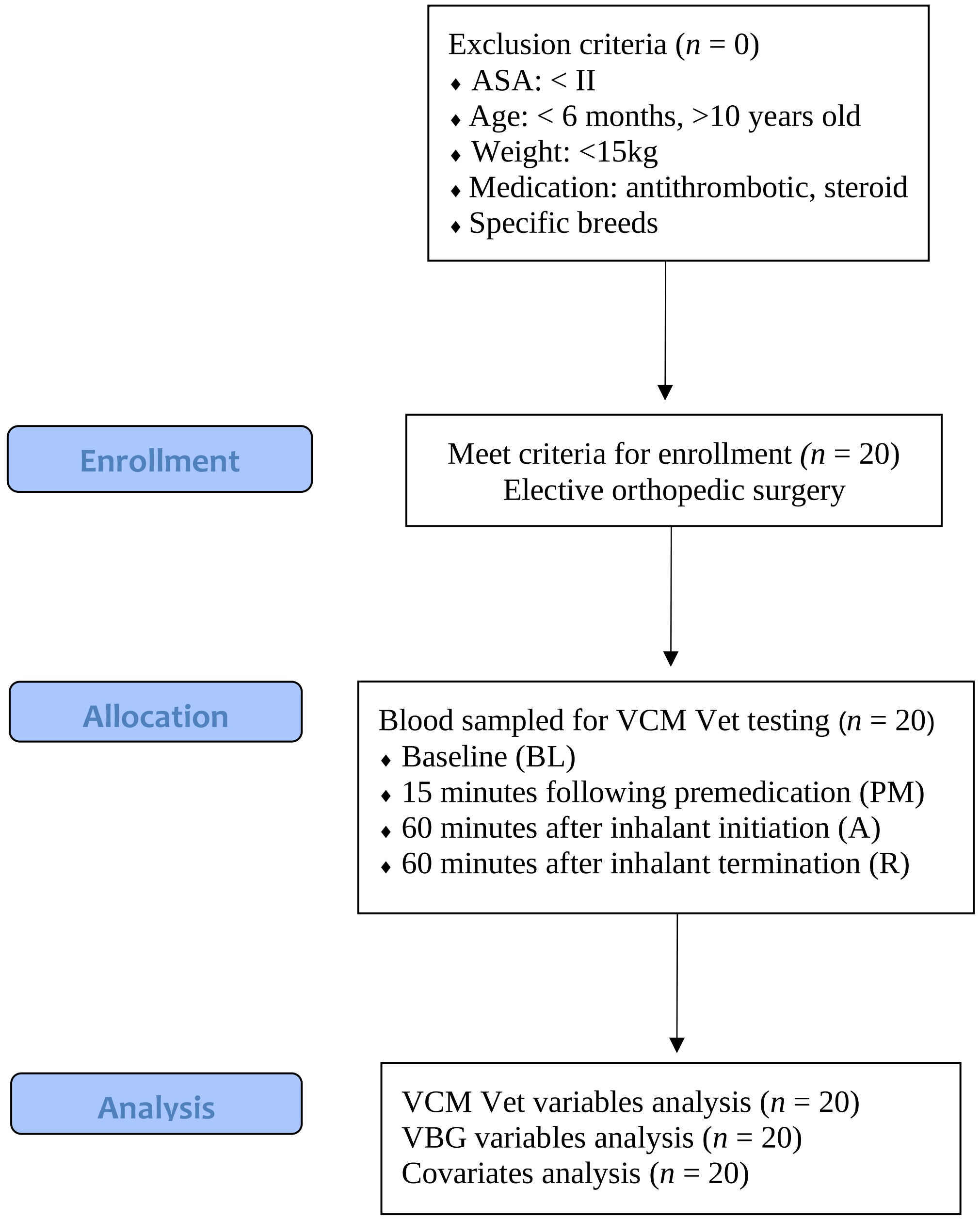

Supplement: Supplementary file 1 — Additional file 1: Appendix A. Rubric consisting of representative Viscoelastic Coagulation Monitor Vet (VCM Vet) tracings for hypercoagulability, normocoagulability, hypocoagulability, and hyperfibrinolysis in healthy 20 dogs undergoing anesthesia and elective orthopedic surgery. Appendix B. Flow diagram on data collection process. [file 12917_2022_3442_MOESM1_ESM.pdf]
